# SARS-CoV-2 is not detectable in cervicovaginal secretions from women with active COVID-19 infection- a pilot study

**DOI:** 10.5935/1518-0557.20210103

**Published:** 2023

**Authors:** Pankush Gupta, Aashish Choudhary, Deepankar Srigyan, Neena Malhotra

**Affiliations:** 1 All India Institute of Medical Sciences- New Delhi, India

**Keywords:** cervicovaginal secretion, COVID-19, SARS-CoV-2

## Abstract

**Objective:**

To detect the shedding of SARS-CoV-2 in cervicovaginal secretions of women with active COVID 19 infection.

**Methods:**

A cross-sectional study from a COVID facility including women aged 20-45 years with active COVID-19 infection, cervicovaginal secretions were collected from cervix and posterior fornix using dacron swab within 7 days of symptom onset or 5 days of nasopharyngeal rRT-PCR test positivity in asymptomatic women. Cervicovaginal samples of women with mild symptoms were tested using rRT-PCR for SARS-CoV-2.

**Results:**

SARS-CoV-2 was not detected in cervicovaginal secretions of any of the 11 women included in the study.

**Conclusions:**

SARS-CoV-2 does not shed in the cervicovaginal secretions of women with mild COVID 19 infection, ruling out sexual and vertical transmission of virus in mild and asymptomatic disease.

## INTRODUCTION

Severe acute respiratory syndrome coronavirus 2 (SARS-CoV-2) has severely affected the world. The disease pathogenesis has been well elucidated but despite the various acceptable modes, transmission via sexual contact remains unresolved. The virus binds to Angiotensin converting enzyme 2 (ACE 2) receptors, leading to the disease manifestations (Yue et al., 2020). ACE 2 receptors have been isolated from various body parts, including the testis - which formed the basis for viral transmission in the semen (Mohseni et al., 2020). However, studies to date have found otherwise (Holtmann et al., 2020). SARS-CoV-2 in vaginal secretions has also been studied with conflicting results (Cui et al., 2020; Qiu et al., 2020; Aslan et al., 2020; Schwartz et al., 2021). There is of ACE 2 receptors expression in the endometrium, suggesting the possible shedding of virus in cervicovaginal secretions (Henarejos-Castillo et al., 2020). Previous viral shedding of other viruses from the coronavirus family has been seen in vaginal secretions, as well as their transmission to the fetus (Gagneur et al., 2008). Given the structural homology of the SARS-CoV-2 with other coronaviruses, it may be hypothesized that it sheds in cervicovaginal secretions, and it may play a role in sexual and vertical transmissions. Hence, we planned this study to assess the viral shedding in cervicovaginal secretions that could throw light on other modes of virus transmission including, sexual and vertical.

## MATERIALS AND METHODS

This cross-sectional study was conducted between October 2020 - December 2020 at a tertiary care hospital that was converted into a dedicated COVID hospital during the pandemic. The women included were between 20-45 years of age with SARS-CoV-2 infection, confirmed by RT-PCR on nasopharyngeal swabs and mild symptoms within 7 days of onset. We also included the asymptomatic women within 3-5 days of testing positive on RT-PCR. We excluded women with moderate-severe disease, with comorbidities or during their menstrual period. Women after informed written consent underwent a per speculum examination by trained personnel. Cervicovaginal secretions were collected with two dacron swabs, one inserted into the cervix and rotated 360 degrees for 3-4 times and another inserted into the posterior fornix to collect vaginal secretions. Adequate PPE was given to those handling and transporting the cervicovaginal samples.

Collected cervicovaginal swab specimens were inoculated into 3 ml of Viral Transport Medium (HiMedia laboratories, India), labelled with the patient's information and transported in an icebox immediately to the dedicated SARS-CoV-2 detection laboratory, at the All India Institute of Medical Sciences, New Delhi.

### Laboratory procedure

All the specimens were processed in a class II, Type A2 biological safety cabinet with biosafety level-2 (BSL2) work practices. The transport tubes were briefly vortexed to homogenize the specimen with the viral transport medium. After removing the swab from the transport medium, the samples were centrifuged at 3000 rpm for 5 minutes. Then the transport tube contents were aliquoted in two 1 ml aliquots in pre-labelled screw-capped storage vials and stored at -80°C until testing.

RNA extraction and Real-Time RT-PCR Assay:

RNA was extracted from the cervicovaginal swab specimens using the QIAamp Viral RNA Mini Kit (Qiagen, Cat. No. 52906). RNA was extracted from 200 µl of the specimen and eluted in 30 µl of elution buffer provided with the extraction kit. The RNA was reversely transcribed and amplified using an FDA-EUA approved, ICMR-NIV multiplex single tube SARS-CoV-2 RT-PCR assay (India) in Agilent AriaMx Real-Time PCR system. The results were interpreted as per the manufacturer's instruction provided within the PCR kit.

## RESULTS

Our study had 11 sexually active females admitted with mild disease. The baseline characteristics are summarized in Table 1. The virus was not detectable in the cervicovaginal secretions of these women. Husbands of four out of these 11 females were also positive and their semen samples were also collected for and tested for virus shedding, which also did not detect the virus.

**Table 1.** Baseline characteristics of enrolled participants (n=11).

Variable	
Age (years) (Mean±SD)	34±5.65
BMI (Kg/m^2^) (Mean±SD)	22.9±1.33
Duration of symptoms (days) (Median)	2
Symptomatic (%)	63.6
Time from symptom onset to cervicovaginal swab (days) (Mean±SD)	4.0±0.89
Time from testing to cervicovaginal swab (days) (Median)	3

BMI - Body Mass Index, SD- Standard deviation.

## DISCUSSION

Our study intended to define other modes of SARS-CoV-2 virus transmission. In our study cohort of women in the active phase of disease, no viral shedding was seen in the cervicovaginal secretions; thus, suggesting against female to male transmission of virus through sexual contact, and from mother to child during vaginal delivery.

Coronavirus attaches to ACE 2 receptors in different organs through a spike protein, causing its fusion with cell membranes and endocytosis (Henarejos-Castillo et al., 2020). A TMPRSS protease cleaves the S protein, which further causes its binding to ACE2 receptors. Other proteases, such as the Cathepsin B , L , FURIN, MX1, Basigin (BSG) have also been shown to play a role in increasing Coronavirus infectivity. A study by Henajeros-Castillo et al. tried to find the expression of these various proteins in the endometrium of healthy patients to assess the vulnerability of the endometrium to this virus, and they found high gene expression of TMPRSS4, CTSL, CTSB, FURIN, MX1 and BSG in the endometrium. Although ACE2 expression was low, it increased towards the implantation window and with age, suggesting endometrial susceptibility to CoV-2 more in the elderly with possible cervical shedding (Henarejos-Castillo et al., 2020). The SARS CoV-2 Genome is 89% like that of other coronaviruses, such as the HCoV-229E, which have already been proven to infect the vagina and transmit sexually, and even cause vertical transmission (Gagneur et al., 2008). Therefore, we can speculate that the transmission potential of CoV-2 will be like that of other viruses of the family. These findings are refuted by a recent study by Cui et al., where they pointed out that the ACE2 receptors are not expressed in cervical and vaginal tissues, so females do not transmit the virus sexually or during delivery to the baby through vaginal secretions (Cui et al., 2020). Another study by Qiu et al. in postmenopausal women with severe disease, also evaluated vaginal secretions obtained between 17-49 days after infection but did not find any positive sample (Qiu et al., 2020). Aslan investigated 12 pregnant women with moderate disease for positivity in vaginal secretions and did not find the virus in the secretions (Aslan et al., 2020). Only one study, by Schwartz et al., detected the SARS-CoV-2 in vaginal secretions with acute infection in mild disease in 2/35 women, one premenopausal and one postmenopausal (Schwartz et al., 2021).

The present study has some limitations, such as the small sample size, non-inclusion of postmenopausal women and women with severe disease. Despite these limitations, the present study surely provides evidence concerning the absence of the virus in cervicovaginal secretions and no risk of fetal affliction in mild disease. Future studies should aim to include more patients with severe disease to reach a conclusion.

## CONCLUSION

No shedding of SARS-CoV-2 virus occurs in cervicovaginal secretions in women with mild disease, pointing against sexual or vertical transmission of the virus.
